# Impact of COVID-19 pandemic on population-based cancer screening: Interrupted Time Series Analysis

**DOI:** 10.1093/eurpub/ckac131.081

**Published:** 2022-10-25

**Authors:** JDG Pereira Godinho Simões, P Pita Ferreira, C Pinto de Carvalho, P Pinto Leite, A Peralta-Santos

**Affiliations:** 1 Directorate of Information and Analysis, Directorate-General of Health, Lisbon, Portugal; 2 Public Health Unit of Almada-Seixal, Regional Health Administration of Lisbon and Tagus Valley, Almada, Portugal; 3 Public Health Unit of Oeste Norte, Regional Health Administration of Lisbon and Tagus Valley, Caldas da Rainha, Portugal; 4 Public Health Unit of Litoral Alentejano, Regional Health Administration of Alentejo, Santiago do Cacém, Portugal; 5 Department of Global Health, University of Washington, Seattle, Washington, USA; 6 National School of Public Health, NOVA University of Lisbon, Lisbon, Portugal

## Abstract

Early evidence suggests that the COVID-19 pandemic may have reduced the proportion of individuals submitted to cervical, colorectal and breast cancer screening. However, the recovery from the pandemic impact was very heterogeneous. We aim to explore the impact of the pandemic on cancer screening and estimate the time to recover lost screening opportunities in Portugal. We used an interrupted time series to analyze the impact of the pandemic on the implementation of cancer screening. The study population was the eligible individuals screened for cervical, colorectal and breast cancer by month and health region between 2018 and 2021. We used Poisson regression with health region random effects to estimate the trend before and after the first lockdown (March 2020) and the impact of the first lockdown. We predicted the counterfactual evolution without a pandemic to estimate lost screening opportunities. The first lockdown resulted in 93,1% (95%CI 92,9-93,2), 89,4% (95%CI 89,2-89,5) and 84,1% (95%CI 83,8-84,3) decrease in the proportion of expected cervical, colorectal and breast cancer screening tests. Nonetheless, we document an increased trend difference between pre and post lockdown of 6,0% (95%CI 5,9-6,0) and 5,3% (95%CI 5,3-5,4), 3,7% (95%CI 3,6-3,7) per month. However, by December 2021, there are still many lost screening opportunities due to the pandemic - 293k cervical cytology tests (42,2% less than expected), 247k fecal occult blood tests (28,7%) and 388k mammograms (38,4%). The first lockdown resulted in an abrupt decrease in cancer screening. However, we document an increase in the cancer screening trend after the pandemic. Nevertheless, there are still considerable lost screening opportunities after 2 years.

**Key messages:**

• The pandemic caused a massive disruption in cancer screening. Although there was an increase in screening trends after the first lockdown, 2 years later, many lost screening opportunities remain.

• Population-based screenings need to increase the outputs to account for lost screening opportunities due to the pandemic.

